# 

**Published:** 1840-08

**Authors:** 


					﻿THE
WESTERN JOURNAL
OF
MEDICINE AND SURGERY.
COLLABORATORS.
Edward II. Barton, M. D. Profes-
sor of the Theory and Practice of
Medicine, in the Medical College of
Louisiana.
William J. Barbee, M. D. George-
town, Ky.
George W. Bayless, M. D. Dissector
for the Pathological Cabinet of the
Louisville Medical Institute.
A- II. Buchanan, M. D. Columbia,
Tennessee.
Charles Caldwell, M. D. Professor
of the Institutes of Medicine and
Medical Jurisprudence, in the Lou-
isville Medical Institute.
Samuel A. Cartwright, M. D. Mis-
sissippi.
A. Clapp, M. D. New Albany, In-
diana.
Jedediah Cobb, M. D. Professor of
Anatomy, in the Louisville Medical
Institute.
John Esten Cooke, M. D. Professor
of the Theory and Practice of Medi-
cine, in the Louisville Medical In-
stitute.
Samuel D. Gross, M. D. Professor of
Surgery in the Louisville Medical
Institute.
John Hardin, M. D. Munfordsville,
Ky.
John P. Harrison, M. D. late Profes-
sor of Materia Medica,in the Cin-
cinnati College.
John F. Henry, M. D. Bloomington,
Illinois.
Samuel P. Hildreth, M. D. Marietta,
Ohio.
Samuel Hogg, M. D. Nashville, Ten-
nessee.
M. Z. Kreider, M. D., Lancaster,
Ohio.
Moses L. Linton, M. D. Springfield,
Kentucky.
Henry Miller, M. D. Professor of
Obstetrics and the Diseases of Wo-
men and Children, in the Louisville
Medical Institute.
John W. Monette, M. D. Washing-
ton, Mississsippi.
Henry Perrine, M. D. Florida.
Samuel B. Richardson, M. D. Lou-
isville, Ky.
John L. Riddell, M. D. Professor of
Chemistry in the Medical College
of Louisiana.
Landon C. Rives, M. D. late Pro-
fessor of Obstetrics, in the Medical
Department of the Cincinnati Col-
lege.
Charles W. Short, M. D. Professor
of Materia Medica, in the Louisville
Medical Institute.
G. Troost, M. D. Professor of Chem-
istry and Mineralogy, in the Uni-
versity of Nashville.
Amasa L. Trowbridge, M. D. Pro-
fessor of Surgery, Willoughby Uni-
versity, Ohio.
John A. Warder, M. D. Cincinnati,
Ohio.
William Wood, M. D. Cincinnati,
Ohio.
The Western Journal of Medicine and Surgery is published monthly by
the undersigned, -at the corner of Main and Fifth streets, Louisville, at $5 per
annum, payable in advance. Each number contains from 80 to 84 pages making
two volumes in the year of about 500 pages.
Contributions to its pages by the Physicians of the Valley of the Mississippi,
addressed to the Editors, are respectfully solicited.
Letters on business, to be addressed, postage paid, to the publishers.
July 25, 1840.	PRENTICE & WEISSINGER.
				

